# Recent advances in population genetics of ectomycorrhizal mushrooms *Russula* spp.

**DOI:** 10.1080/21501203.2015.1062810

**Published:** 2015-06-24

**Authors:** Pengfei Wang, Ying Zhang, Fei Mi, Xiaozhao Tang, Xiaoxia He, Yang Cao, Chunli Liu, Dan Yang, Jianyong Dong, Keqing Zhang, Jianping Xu

**Affiliations:** a Laboratory for Conservation and Utilization of Bio-Resources, and Key Laboratory for Microbial Resources of the Ministry of Education, Yunnan University, Kunming, Yunnan, PR China; b Yunnan Institute for Tropical Crop Research, Jinghong, Yunnan, PR China; c Department of Biology, McMaster University, Hamilton, Ontario, Canada

**Keywords:** *Russula*, ecological strategy, genet, isolation by distance, population genetics

## Abstract

The mushroom genus *Russula* is among the largest and morphologically most diverse basidiomycete genera in the world. They are broadly distributed both geographically and ecologically, forming ectomycorrhizal relationships with a diversity of plants. Aside from their ecological roles, some *Russula* species are gourmet mushrooms. Therefore, understanding their population biology and fundamental life history processes are important for illustrating their ecological roles and for developing effective conservation and utilization strategies. Here, we review recent population genetic and molecular ecological studies of *Russula*. We focus on issues related to genet sizes, modes of reproduction, population structures, and roles of geography on their genetic relationships. The sampling strategies, molecule markers, and analytical approaches used in these studies will also be discussed. Our review suggests that in *Russula*, genets are typically small, local recombination is frequent, and that long-distance spore dispersal is relatively uncommon. We finish by discussing several long-standing issues as well as future trends with regard to life history and evolution of this important group of mushrooms.

## 1. Introduction

Ectomycorrhizal fungi (EMF) are known as mutualistic symbiosis partners associated with host plants. In this relationship, fungi help plants obtain minerals and water as well as enhance plants’ resistances against pathogen infections and environmental stresses (Schützendübel and Polle 2002; Morgan et al. 2005; Benito and González-Guerrero 2014). In return, host plants supply carbohydrates and unique ecological niches to these fungi (Hobbie 2006; Smith and Read 2008). Globally, a large percentage of terrestrial plants form this type of plant–fungal mutualism. Therefore, in natural ecology systems, ectomycorrhizal symbiosis is one of the most ubiquitous phenomena that play important roles in accelerating nutrient and mineral cycling and in maintaining ecosystem stability (Read and Perez-Moreno 2003; Smith and Read 2008).

**Figure 1.**
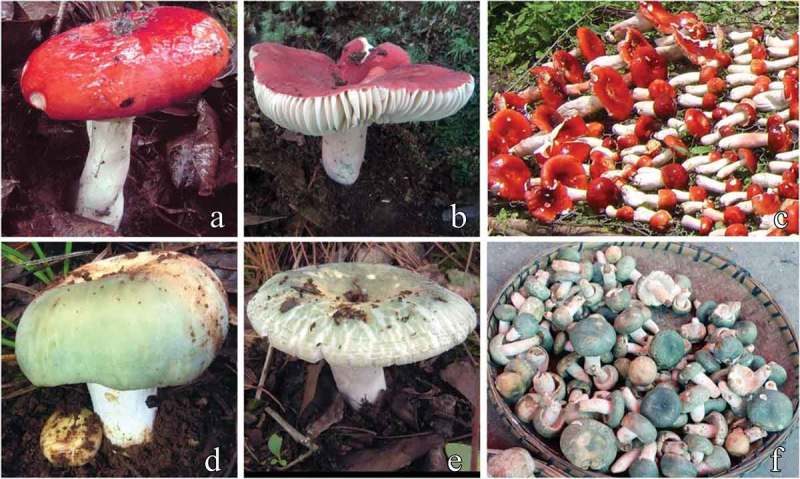
Two ecologically and economically important *Russula* species in southwestern China. (a) An immature and (b) a mature sporocarp of the *Russula griseocarnosa* species complex. (d) An immature and (e) a sporocarp of the *Russula virescens* species complex. (c) and (f) were taken from mushroom markets in Yunnan. Figures (a), (b), (d), and (e) are provided by Dr. Feng Bang (Key Laboratory of Biodiversity and Biogeography, Kunming Institute of Botany, Chinese Academy of Sciences).

Many basidiomycetes are EMF. Several most specious basidiomycete families including Russulaceae, Thelephoraceae, Boletaceae, and Amanitaceae are EMF-rich (Gardes and Bruns 1996; Tedersoo et al. 2010; Lancellotti and Franceschini 2013). Among EMF, the genus *Russula* is one of the most broadly distributed. Globally, about 750 species have been recorded in this genus (Kirk et al. 2008). Some *Russula* species (e.g., *Russula nigricans* and *Russula pectinatoides*) are found in several continents (Eberhardt 2002; Miller and Buyck 2002; Shimono et al. 2004; Ashkannejhad and Horton 2006; Palmer et al. 2008; Yin et al. 2008; Jones et al. 2012; Osmundson et al. 2013; Park et al. 2014), whereas others show geographic specific distributions (e.g., *Russula brevipes* is mainly distributed in North America, *Russula ochroleuca* is mainly distributed in Europe, and *Russula discopus* is dominated in tropical zone) (Bergemann and Miller 2002; Eberhardt 2002; Miller and Buyck 2002; Riviere et al. 2007; Kellner et al. 2009; Kottke et al. 2010; Kleine et al. 2013; Osmundson et al. 2013). Over the past decade, biodiversity surveys using new molecular methods and/or analyzing previously under-surveyed territories and ecological niches have led to the discovery of many new species in this genus, such as *Russula changbaiensis* (northeast China), *Russula tsokae* (Sikkim, Himalayas), *Russula galbana* (Australasia), and *Russula caeruleoanulata* (west Africa) (Lebel and Tonkin 2007; Douanla-Meli and Langer 2009; Das et al. 2010; Li et al. 2013). Recent studies found *Russula* spp. can form ectomycorrhizae with many plant species in a broad range of plant families, including Leguminosae, Fagaceae, Dipterocarpaceae, and Pinaceae (Horton and Bruns 2001; Bergemann 2002; Bergemann et al. 2006; Riviere et al. 2006; Dickie and Moyersoen 2008; Xie et al. 2010). Therefore, the findings on the distribution, abundance, and host ranges of *Russula* show that this genus likely plays significant roles in the global forest ecosystem. Figure 1 shows two typical *Russula* species widely distributed in southwestern China. Aside from their ecological roles, a large proportion of *Russula* are edible, regarded as excellent sources of food and nutrients and contribute significantly to the local economy in some parts of the world, as shown in Figure 1(c) and 1(f) (Boa 2004; Li et al. 2010; Cao et al. 2013).

Morphologically, *Russula* is easily distinguished from other common mushrooms by several recognizable features: a white to dark yellow spore print, free white gills and without partial veil or volva tissue on the stipe, and brittle fleshy basidiocarp due to the presence of sphaerocysts. There is no milky latex, as is commonly released by species in *Lactarius* (Singer 1986). However, the macro-morphological features such as cap color can be highly variable within a species and there are relatively few features to distinguish many closely related species within this genus. Indeed, given its specious nature, *Russula* has been one of the most difficult genera for fungal systematics and taxonomy (Singer 1986; Miller and Buyck 2002).

Over the past decades, achievements in plant ecology have accelerated the development of fungal ecology (Harper 1977; Grime 1977). Some concepts and ecology theories in plant ecology are directly adopted by mycologists (Andrews 1992; Dahlberg and Stenlid 1994; Dahlberg 1997). One of the most popular hypotheses divided the lifestyles of EMF into “early-stage fungi” and “late-stage fungi.” “Early-stage” EMF are considered as pioneer colonists in early successional forest. Typical ecological characteristics for an early-stage EMF or EMF communities include a high number of genotypes, small and short-lived genets, low-level of required nutrient, and the release and dispersal of meiotic spores (De La Bastide et al. 1994; Muller et al. 2004). On the other hand, “late-stage” EMF are known to have large and long-lived genets with high level of nutrient requirement and mycelia expansion to spread in mature and undisturbed forests. Therefore, “late-stage” EMF community would have low level of genotypic diversity (Amend et al. 2009; Fiore-Donno and Martin 2001). However, this division is facing challenges. Along with the increasing number of investigated EMF species, some exceptions have been found to these divisions, such as undisturbed old-stands present “early-stage” EMF species with a large genet size (Gherbi et al. 1999; Kretzer et al. 2004). Indeed, many elements may affect the EMF ecological strategy in natural conditions, such as biological characteristics of the species, strain genotype, nutrient level, and other biotic and abiotic factors. The challenges to the dichotomous EMF division have mainly come from population genetic and molecular ecology data that are providing novel insights into specific EMF life histories (Riviere et al. 2006; Li et al. 2010; Douhan et al. 2011; Cao et al. 2013; Kleine et al. 2013). In this paper, we review recent population genetics and molecular ecology of *Russula* spp.

## 2. Fine-scale genetic analyses of field samples

In a fine-scale investigation, genetic structure analyses among individual fruiting bodies from a certain species at a specific location can help us better understand its distribution pattern, reproductive biology, and even ecological strategy (Dahlberg and Stenlid 1994; Bergemann and Miller 2002; Grebenc et al. 2009). Here, individually mapped fruiting bodies from the field are the typical units of analyses. Basic questions related to a genetic individual (i.e., genet) can be directly inferred. A genetic individual is commonly defined as the collection of vegetative mycelia derived from a single mating event. Specifically, the size and persistence of individual genets, the relationship among adjacent genets, and their density and spatial distribution patterns are among some of the basic parameters that these studies can assess.

Species in the genus *Russula* have been traditionally considered as “late-stage” EMF species since people usually found them in mature stands in temperate forests, representing a major proportion of basidiocarps in those forests (Keizer and Arnolds 1994; Redecker et al. 2001; Bergemann and Miller 2002). Another feature supporting its “late-stage” EMF status is that basidiospores of *Russula* are difficult to germinate in laboratory conditions (Nara 2009). However, whether the low germination rate in the lab reflects its natural condition remains to be determined. Indeed, several fine-scale studies contradicted the “late-stage” life history view of *Russula*. For example, in *Russula vinosa*, the genet size was estimated at no more than 1 m because each sporocarp represented a unique genet (Liang et al. 2004). In another species *Russula cremoricolor*, except one genet represented by two sporocarps separated by about 12 m, most genets were less than 1.1 m in diameter (Redecker et al. 2001). Bergemann and Miller (2002) also found that the size of a majority of genets in *R. brevipes* were less than 3 m, with a few exceptions of up to 18 m. A follow-up study with *R. brevipes* revealed a similar pattern: lots of small genets and very few large ones, with the largest at about 25 m (Bergemann et al. 2006). In southern India, one research investigated the genet size of *R*. subsect. Foetentinae and showed sporocarps of the same genets concentrated in the area of diameter less than 5 m, while some may be as big as 70 m (Riviere et al. 2006). The authors emphasized that those large genets were abnormal because there were no connecting basidiocarps over those distances. This situation is also observed in the aforementioned studies. Taken together, these investigations suggested that *Russula* spp. have relatively small genet sizes (less than 5 m), challenging the “late-stage” definition of *Russula*.

While most genets in *Russula* were small, observations of a few large genets do require explanations. These large genets suggest that some genotypes can expand asexually by mycelia growth and/or persisted over a long period of time below ground. However, other factors could also contribute to the inferred large size for a small number of genets, including human activities, transportations by fungusivore, the physical movement of soil and/or other events that may act to repeatedly inoculate the same genotype in the forest. Additional factors including experimental factors, such as mistaken specimen identity and a limited power of the molecular markers to separate closely related genotypes (see also molecular markers later), may also contribute to the identifications of identical genotypes from distant locations. Despite these potential problems, the current results suggest that the reproductive biology of *Russula* and other ECM fungi likely vary among species of the same genus and among ecological niches of a specific species.

While species and genotypes may differ in their persistence in nature, the role of broad-scale ecological factors cannot be ignored. For example, old tropical rainforests have shown to have larger genets of selected EMF than those in young, temperate forests. In two species belonging to a different ectomycorrhizal group, *Suillus bovinus* and *Suillus variegatus*, genets in older forests are fewer but bigger than those in younger forests (Dahlberg and Stenlid 1994; Dahlberg 1997). However, there is no definitive proof that the age of tree stand determines the genet size of the same genotype for any *Russula* species. Further studies should focus on specific *Russula* species in different-age forests but with similar environmental conditions.

Inbreeding coefﬁcient (*F_IS_*) has traditionally been used as an indicator to infer reproductive biology not only in EMF but also in other species (Wright 1922; Slate et al. 2004). This parameter relates to the differences in the amounts of observed and expected heterozygosity in natural populations and can be used to infer reproductive biology within subpopulations. Positive *F_IS_* values mean deﬁciency of observed heterozygotes compared with that expected under random mating and inbreeding within subpopulations. In contrast, a negative value means excess of observed heterozygotes over that expected from random matings among local genetic neighborhoods. Bergemann and Miller (2002) investigated population structures of two separated populations of *R. brevipes*. The Sitka spruce location had a negative inbreeding coefﬁcient (−0.080), which suggested frequent outbreeding and recombination and heterozygous advantage in this population. In contrast, a positive *F_IS_* value (0.151) obtained in a location of the lodgepole pine suggested inbreeding and outbreeding depression. It should be noted that there was limited statistical support for either population to show significant departure from random mating. Despite the lack of statistical support, there seemed evidence for changes in reproductive biology of *R. brevipes* associated with tree stand age since the lodgepole pine forest (100 years) is much older than the Sitka spruce forest (40–60 years). As mentioned before, stand age was correlated to genet size of two *Suillus* species. However, genet sizes at both the Sitka spruce forest and the lodgepole pine forest are quite small. In addition, it is presently unknown whether the observed difference was due to tree-age and host plant species.

In diploid organisms, multilocus allelic data can be used to measure recombination. One of the most common tests is called Hardy–Weinberg equilibrium test. Under some assumptions, such as a large population size, no gene flow, no selective pressure on involved markers, and no emerging mutations, allele and genotype frequencies in a population will remain constant from generation to generation. Generally, researchers assume that target population meets these conditions and use Hardy–Weinberg equilibrium test results to infer population structure and reproductive behavior. Bergemann et al. (2006) used six SSR loci to detect the population structure of *R. brevipes* in an oak/pine woodland. Except one loci that showed obvious heterozygote deﬁciencies in several datasets, most other loci were consistent with Hardy–Weinberg equilibria. The association of alleles at two or more loci was inferred by genotypic equilibrium (an analogous calculation of linkage disequilibrium test). Excluding the specific locus, no significant allele association was found among other loci, suggesting random recombination in natural *R. brevipes* population (Bergemann et al. 2006). For these two tests, it should be noted that sample size should be sufficient. A small sample size can result in a lack of power to reject the null hypothesis of random recombination. To detect whether the sample size was sufficient, a power test is usually recommended to determine the saturation point between sample size and allelic diversity (Bergemann et al. 2006). Above two studies imply evidences for random mating in some fine-scale natural populations of *R. brevipes*. This indicated frequent sexual reproductions and genetic recombination in this “late-stage” species.

The persistence of genets also can help us understand the biology of EMF. Recent studies indicated contradictory conclusions of genet persistence for *R. brevipes*. In a 100-year-old lodgepole pine forest, Bergemann and Miller (2002) obtained sporocarps which had the same genotype as vouchers collected 11 years before. This result suggested *R. brevipes* could persist for a long period in their locality. However, another study showed that, in an oak/pine woodland, 82% genets of *R. brevipes* between two successive seasons were different (Bergemann et al. 2006). The high replacement rate of genotypes suggested frequent sexual reproductions and short genet persistence. Similar contradictory results were observed in *Hebeloma cylindrosporum* (Gryta et al. 1997, 2000; Guidot et al. 2002). At present, the mechanisms for such conflicting results are not known. It is likely that fungal, plant host, environmental, and human sampling factors could all have contributed to the observed differences between sites and studies. For example, some genets may be perennial below ground but each of them may not fruit every year.

Indeed, the life cycle of EMF is highly dependent on host plants. EMF sporocarps tend to form around host trees rather than randomly distributed. Riviere et al. (2006) found that the spatial distribution of *R*. subsect. Foetentinae basidiocarps was highly aggregated with about 60% of them located at a distance of less than 1 m from the nearest one. Bergemann and Miller (2002) also found that sporocarps of *R. brevipes* fruited in clumped aggregations tended to have the same genotype. Because *Russula* typically have small genets, they were supposed to form by frequent sexual reproductions. The dispersal of spores can have a significant impact on genotype distribution at a local level. Thus, the spatial distribution pattern of genets can be used to infer the gene flow level in a fine-scale research. The negative correlation between genotypic similarity and geographic distance between genets indicated restricted gene flow due to short-distance spore dispersal, whereas no spatial correlation means unrestricted gene flow (unrestricted spore dispersal) (Kretzer et al. 2005; Dunham et al. 2006). Liang et al. (2004) found sporocarps of *R. vinosa* fruited as clumped patches. The radius of each patch was about 20 m and pairwise sporocarps closer than 10 m had a significantly higher genetic similarity than those further away. In addition, the high-genetic-similarity group tended to have small clumps with high fruiting body density, whereas the low-genetic-similarity group tended to have large clumps with low fruiting body density (Liang et al. 2004). The fine-scale analyses suggest gene flow via spore dispersal is generally quite limited.

## 3. Large-scale genetic analyses

Different with the fine-scale studies, large-scale genetic analyses have mainly focused on population subdivision, isolation by distance and gene flow over long distances in target species. Like pollens in plants, mushroom spores can be dispersed by wind (called anemophily). However, the role of wind-mediated gene flow in large-scale population genetic studies is still controversial. Several studies have assessed the aerodynamics of spore transmission in air. In nature, air-mediated dispersal pattern of spores seem to follow a Poisson distribution pattern with most spores land nearby their parental sporocarps (Kuparinen et al. 2007; Galante et al. 2011). While fine-scale population genetic analyses suggested predominantly localized spore dispersal, it does not exclude the possibility of occasional long-distance dispersal. Because certain geographic barriers such as mountain and river systems, or long distances, can completely disrupt the extension of hyphal networks, any observed gene flow and allele sharing among distinct geographic populations could have been contributed by spore dispersals.

Cao et al. (2013) used sequences at five gene fragments to infer the structure and gene flow among geographic populations of a *Russula virescens* ally in southwestern China (the largest separation distance between populations was about 500 km). Results showed all five loci each had at least one haplotype distributed in most local populations. More importantly, the AMOVA test showed that over 90% of the observed genetic variation was found within individual local populations, consistent with significant gene flow among populations. However, while some pairs of populations showed significant genetic differentiation, the relationship between pairwise population genetic differences (*F_ST_*) and geographical distances was statistically insignificant. The allelic association tests among loci indicated that population of *R. virescens* in southwestern China was randomly mating.

Different from *R. virescens*, phylogeographic analyses among distinct populations of the *Russula griseocarnosa* species complex in southwestern China showed significant genetic differentiation, with three distinct geographic regions each having a different genetic population (Li et al. 2010). However, evidence for limited spore dispersal and gene flow between some of the local and regional populations were also found. Both of the above studies investigated geographic populations in southwestern China, mainly in Yunnan province. This region has many unusual characteristics such as big mountains, deep gorges, and numerous streams and rivers. Similar observations have also been made by Bergemann and Miller (2002) in North America and by Kleine et al. (2013) in Africa. Specifically, there was little overlap in allele frequencies between two populations of *R. brevipes* in North America separated by the Rocky Mountains (Bergemann and Miller 2002). In addition, Kleine et al. (2013) identified genetic divergence among disjunct populations on the African continent and Madagascar for three species *R. discopus, Russula pseudocarmecina*, and *Russula ochraceorivulosa*. Together, these studies suggest that long-distance spore dispersal is generally limited in *Russula* species and if it exists, it were likely achieved through multiple short distances that could reach intermediate and long distances.

## 4. Sampling strategies and molecular markers

In statistics, sampling usually takes the advantage of a subset of individuals from a statistical population to represent and estimate characteristics of the whole population (Doherty 1994; Pagano et al. 2000). For fine-scale population genetics research, a sampling strategy that collects all sporocarps is generally required for genet definitions and spatial distribution analyses. The most comprehensive sampling in such a situation requires the location to be continuously monitored over one or more complete fruiting seasons. Because fruit body production depends on many factors and can vary from one year to another, to ascertain the genet persistence over time and identify community temporal changes in a certain habitat, comprehensive sampling over many years may be required (Trudell and Edmonds 2004; Bergemann et al. 2006; Durall et al. 2006). However, researchers should be cautious in matching sampling strategies to their specific research objectives. For example, comparing temporal samples from a specific location between seasons/years, fruiting bodies should not be removed from the field. Removing basidiocarps in one year may severely impact the genotype of the emerging genets and reduce the genotypic diversity in the following successive years. If all fruiting bodies were removed, the probability of obtaining the same genet the following year would increase and that of obtaining recombinant genotypes could decrease, and result in biased conclusions on natural population successions. Instead, a small portion of the cap tissues should be sampled.

In practice, it is often impossible or impractical to analyze all individuals from a population. Selective sampling based on an established set of criteria is often chosen to reduce the workload while still maintaining the sample’s representativeness (Durall et al. 2006; Riviere et al. 2006). When taken selective sampling strategies, the criteria and assumptions should be noted and the results should be discussed with the limitations in mind. For example, Redecker et al. (2001) assumed *a priori* that there were two *Russula* species based on the distinct color of pileus (red-capped for *Russula silvicola* and white-capped for *R. cremoricolor*). However, they found that those two morphological types have identical sequences at the internal transcribed spacer (ITS) regions and the two populations formed a randomly mating population. Here, the genetic results were incongruent with morphological data.

Although obtaining and analyzing all fruiting bodies from a geographic population can be a daunting task for some species, in many cases, the reverse (i.e., insufficient specimen) is often the problem. For example, for edible mushrooms, local residents may pick them, leaving relatively few or none for outside researchers to get samples. Indeed, without help from local residents and institutions, to gather the complete or representative fruiting body samples for genetic analyses in a big area is often very difficult or impossible. In addition, many years may be spent in collecting fruiting body samples when the analyses require samples from many distinct locations. An alternative approach is emerging recently that involves obtaining mycelial samples directly from the soil instead of relying on fruiting bodies. Here, species-specific primers are needed in order to obtain molecular information from the metagenome samples. In addition, since many mycorrhizal fungi are edible, especially for *Russula*, fruiting bodies from mushroom trading markets or village fairs could be an excellent source of genetic materials from which to assess the diversity and genetic relationships among geographic populations. If samples are obtained through markets, it is important to clarify with the mushroom sellers as to where specifically their mushrooms are from. If a mushroom market contained samples from distinct geographic locations that are all mixed together, analyzing such samples could potentially generate erroneous conclusions about the population structure of the specific organism.

A common analysis in population genetic studies is to examine the relationship between genetic distance and geographical distance. It is often assumed that geographically closely related samples should be genetically more similar to each other due to their close breeding relationships. However, to effectively identify the genetic relationships among strains, adequate genetic markers are needed. These markers must have an adequate resolution to distinguish genetic individuals, particularly for a small quadrat. Several types of molecular markers have been used for fine-scale and large-scale population genetic analyses of *Russula* species, including RADP, AFLP, SSR, ISSR, and sequencing of variable genetic loci (Sunnucks 2000; Schlötterer 2004). However, each of the above types of markers has their own unique problems. For example, SSR markers should be used with caution, since “Null allele” may exist (Chakraborty et al. 1992; Callen et al. 1993; Roy et al. 2008). For population genetic analyses, many factors can increase population divergences, and mutations could impact the specificity of SSR primers. The presence of Null alleles will reduce the heterozygosities in populations and make those populations seem inbred (Callen et al. 1993; Roy et al. 2008). RAPD markers can also be impacted by Null alleles that can be further complicated by its poor stability and repeatability. In addition, for PCR fingerprinting-based markers, bands that are indistinguishable based on band size are often scored as belonging to the same locus and the homozygotes cannot be distinguished from heterozygotes for such markers. In contrast, a PCR–RFLP marker is codominant and not affected by Null alleles but their discriminating power is often limited, unless many such markers are used. Sequencing genes can generate abundant polymorphism. However, in diploid organisms, obtaining clean sequences can be difficult if the two alleles contain insertion/deletion polymorphisms. Such problems could be minimized if the designed markers amplify gene fragments that are highly conserved and contain no indel polymorphisms. Due to the potentially different levels of genetic variation within and between populations, it might be necessary to design different molecular markers for different purposes. For example, to identify genetic individuals among fruiting bodies in fine-scale genetic analyses, RAPD and PCR fingerprinting could be highly discriminatory and effective. For large-scale population genetic analyses, PCR–RFLP and PCR-directed sequencing for several highly polymorphic genes might be more desirable.

## 5. Emerging directions

### 5.1. High-throughput sequencing and population genomics

Current high-throughput sequencing technologies are revolutionizing population genetic studies of many organisms. These technologies have reduced sequencing costs significantly and allowed obtaining thousands of sequences relatively affordably for most research labs in a relatively short period of time. In model species, population genomics has become feasible and the new norm. Population genomics mainly focuses on the patterns of evolutionary processes’ impact on genomes through inspecting the differentiation among genomes within and among populations (Luikart et al. 2003; Whitaker and Banfield 2006). Species in genus *Russula* are excellent candidates for population genomic studies on ecological issues. These organisms are broadly distributed EMF and can form extensive associations with numerous types of hardwood trees in various habitats. They play significant roles in plant health. For example, population genomic studies of *Russula* could help solve some historical biogeographic mysteries. The effects of the last ice age on flora and fungal communities have been hotly debated in recent years. The maximum glaciation in the Northern Hemisphere was considered to cover most of North America (Richmond and Fullerton 1986). Icebound earth surface followed by rapid melting would eliminate all surface life forms and significantly impact the landscape. After retreat of the glacier, the ground would be invaded by different groups of organisms. Plants as well as ECM fungi from glacial refugia would be the sources of organisms for these communities. Therefore, measuring the ECM fungal community structure in the Northern Hemisphere can help identify the refugia and better understand species dispersal, species survival, speciation, and the history of ecological niche transitions (Geml et al. 2010, 2012). In this aspect, due to their ubiquity in natural ecosystems, species in the genus *Russula* should be able to provide excellent information from which to understand these issues.

### 5.2. Molecular phylogenetic analysis in population genetics

Population genetics focuses on analyzing the distribution and alteration of allele frequencies within and between populations. The definition of population should reflect the natural mating community of a certain species. Morphological characteristics within and among species in *Russula* can be highly variable and their species identifications are difficult, even for mushroom experts. Although the distribution of allele frequency and pairwise *F_ST_* values can help identify the relationships among populations, population genetic analysis alone is often insufficient to provide a complete historical account of population histories and speciation (Bergemann and Miller 2002). Indeed, because many closely related but distinct species in *Russula* are difficult to distinguish morphologically, morphology-based species identification can provide misleading information on population structures within a species. Instead, other types of analyses such as phylogenetics and tree networks are necessary to help with species delineation and infer population level processes. As shown in Li et al. (2010), the analysis of multilocus sequence data helped define several cryptic species within *R. griseocarnosa*. Only after such cryptic species are identified and sorted can population genetic studies within individual species be accurately performed.

### 5.3. *Russula* in tropical zones

Tropical forests have been traditionally regarded as the Eden of saprophagous organisms but they are poor in EMF (Malloch et al. 1980; Pirozynski 1981). Recent researches investigated the ectomycorrhizal fungal species in tropical zones and found abundant ectomycorrhizal fungal communities below the ground (Buyck et al. 1996; Lee et al. 1997; Riviere et al. 2007; Phosri et al. 2012; Osmundson et al. 2013). Indeed, species in family Russulaceae are now considered one of the most dominant ectomycorrhizal fungal groups in the tropics. Many novel species of *Russula* have been reported recently from tropical zones (Riviere et al. 2007; Phosri et al. 2012; Osmundson et al. 2013). The type genus *Russula* is the most specious genus in family Russulaceae and was first established based on the studies of macro fungi in northern Europe. Historically, many species in *Russula* were established based primarily on European specimens and their taxonomic criteria. The tropical regions contain a high diversity of *Russula* species and some of the criteria used to identify species in northern Europe may not be applicable for identifying those in the tropics. To solve this problem, molecular systematic studies of samples from different geographic origins are needed in order to obtain accurate information about species distribution of *Russula*. Furthermore, by comparing population or community structures between temperate and tropical regions, the investigations of species differentiation, adaptive evolution, and species origin are now feasible.

### 5.4. Below-ground perspectives

While most population genetic studies have focused on analyzing above-ground fruiting bodies, the above-ground sporocarps are unlikely to reflect the below-ground ectomycorrhizal fungal community (Gardes and Bruns 1996; Dahlberg et al. 1997). The appearance of fruit bodies undoubtedly manifests the presence of hyphae of the corresponding species in the soil, but the absence of above-ground fruit bodies do not mean the absence of below-ground fungal populations (Gardes and Bruns 1996; Dahlberg et al. 1997). During sexual mating in basidiomycetes, two monokaryotic cells fuse and form dikaryotic mycelia. Following vegetative growth and if the environmental conditions are favorable, the dikaryotic mycelia can produce fruit bodies. In this process, a crucial factor is the mating compatibility between monokaryons (Moore and Frazer 2002; Billiard et al. 2011). For monokaryotic cells, even if they belong to the same species and are located in the same community close to each other, they can only mate and form fertile mycelia when they have different alleles at the mating type locus (loci) (Casselton and Olesnicky 1998; Moore and Frazer 2002; Billiard et al. 2011). In addition, environmental factors can influence all stages of sexual development. Therefore, sporocarps reflect the achievements of sexual events initiated by either recently mated and fused hyphae or by the dikaryons already existing in the environment. As a result, fruiting is opportunistic and the population structure as revealed by above-ground fruiting bodies will unlikely reflect the below-ground mycelia. To analyze below-ground fungal communities and spatial distributions, researchers should not typically collect soil cores around above-ground fruit bodies. Otherwise, our current knowledge based on such samples could be a biased representation of the below-ground fungal community structure. It has been suggested that some members of Russulaceae fungi never or rarely produce fruiting bodies (Bills et al. 1986). If indeed these species never/rarely reproduce sexually to generate fruiting bodies, their life history, population structure, and ecological function would only be interpreted from below-ground data. At present, there is no report about population structure of *Russula* or any EMF based on below-ground mycelia alone, regardless of whether they produce above-ground fruiting bodies.

For species that frequently produce above-ground fruiting bodies, a central issue is whether population structures represented by below-ground ectomycorrhiza and above-ground fruiting bodies are consistent. At present, the results of such comparisons differ among species. In both *R. xerampelina* and *R. ochroleuca*, their above- and below-ground populations seemed similar to each other while for *R. amoenolens*, there was little similarity between the two components (Agerer 1990; Gardes and Bruns 1996). However, those analyses were focused on below-ground samples from roots of specific plants, not from soil. A comprehensive analysis of soil samples targeting specific species could help us addressing this issue. Furthermore, real-time PCR or meta-genomic analysis could also compare the abundance of different EMF in plant roots/rhizosphere soils and the spatial distribution of hyphae belonging to specific groups of organisms.

Aside from such comparisons, another long-standing issue of ectomycorrhizae is the prevalence and longevity of monokaryotic hyphae in nature. Current researches have focused on dikaryotic mycelia and there was a report that dikaryotic hyphae of *R. brevipes* could maintain a long-term connection with plants (Bergemann and Miller 2002). Other reports also showed the species in genus of *Russula* can immediately form ectomycorrhizae with seedlings in a wildfire-burned forest by surviving hyphae (Horton et al. 1998; Grogan et al. 2000). Regrettably, these studies did not give a clean determination of ploidy of emerging ectomycorrhizae. Theoretically, monokaryotic hyphae should be able to infect plant roots and form ectomycorrhizae, and several *in vitro* infection tests using monokaryotic strains have confirmed this prediction (Tranvan et al. 2000; Costa et al. 2010). Further investigations of spore germinations, infection experiments by monokaryotic hyphae, mycorrhizal synthesis experiment between monokaryotic and dikaryotic hyphae in nature should help us better understand the details of fungal life cycles.

## Disclosure statement

No potential conflict of interest was reported by the authors.
